# Supplementary material for: Programmable adhesion and morphing of protein hydrogels for underwater robots
Source: Nat Commun. 2024 Jan 3;15:195. doi: 10.1038/s41467-023-44564-6 (PMC10764313; doi:10.1038/s41467-023-44564-6)
Supplement: Supplementary file 1 — Supplementary Information [file 41467_2023_44564_MOESM1_ESM.pdf]

Supplementary Information

Programmable adhesion and morphing of protein hydrogels for underwater robots

Sheng-Chen Huang[1], Ya-Jiao Zhu[1], Xiao-Ying Huang[1], Xiao-Xia Xia[1]* & Zhi-Gang Qian[1]*

[1]State Key Laboratory of Microbial Metabolism, Joint International Research Laboratory of Metabolic & Developmental Sciences, and School of Life Sciences and Biotechnology, Shanghai Jiao Tong University, 800 Dongchuan Road, Shanghai 200240, People's Republic of China

*Corresponding authors: xiaoxiaxia@sjtu.edu.cn; zgqian@sjtu.edu.cn.

Supplementary Note 1

The complete amino acid sequence of resilin-like protein R32 expressed from plasmid pET19b-R32:

MGHHHHHHHHHHSSGHIDDDDKHMASGGRPSDSYGAPGGGNGGRPSDSYGAPGG
GNGGRPSDSYGAPGGGNGGRPSDSYGAPGGGNTSGGRPSDSYGAPGGGNGGRPSDS
YGAPGGGNGGRPSDSYGAPGGGNGGRPSDSYGAPGGGNTSGGRPSDSYGAPGGGN
GGRPSDSYGAPGGGNGGRPSDSYGAPGGGNGGRPSDSYGAPGGGNTSGGRPSDSYG
APGGGNGGRPSDSYGAPGGGNGGRPSDSYGAPGGGNGGRPSDSYGAPGGGNTSGG
RPSDSYGAPGGGNGGRPSDSYGAPGGGNGGRPSDSYGAPGGGNGGRPSDSYGAPGG
GNTSGGRPSDSYGAPGGGNGGRPSDSYGAPGGGNGGRPSDSYGAPGGGNGGRPSDS
YGAPGGGNTSGGRPSDSYGAPGGGNGGRPSDSYGAPGGGNGGRPSDSYGAPGGGN
GGRPSDSYGAPGGGNTSGGRPSDSYGAPGGGNGGRPSDSYGAPGGGNGGRPSDSYG
APGGGNGGRPSDSYGAPGGGNTSLEDPAANKARKEAELAAATAEQ

Supplementary Table 1. Compositional features and thermally responsive properties of the hydrogels[a]

Hydrogel type[b]	Silicotungstic acid-to-R32 molar ratio	Water content (%)	T_s (°C)	T_i (°C)
R32-SiW	6.4 ± 0.1	22.7 ± 1.2	20.4 ± 0.3	23.7 ± 0.2
R32-5%-SiW	6.0 ± 0.1	21.5 ± 1.5	21.2 ± 0.2	23.8 ± 0.2
R32-15%-SiW	5.4 ± 0.1	20.6 ± 1.7	22.5 ± 0.4	43.5 ± 0.4
R32-30%-SiW	4.9 ± 0.2	19.1 ± 0.8	26.7 ± 0.3	52.7 ± 0.5

[a]Data are presented as mean ± s.d. of $n = 3$ independent samples, and are representative of two independent experiments. Note that T_s and T_i represent the softening temperature of the hydrogel, and the temperature at which the hydrogel initiated adhesion. Source data are provided as a Source Data file.

[b]The hydrogels were fabricated by complexation of silicotungstic acid (SiW) with resilin-like protein R32 with prior di-tyrosine crosslinking degrees at 0, 5%, 15%, and 30%, respectively.

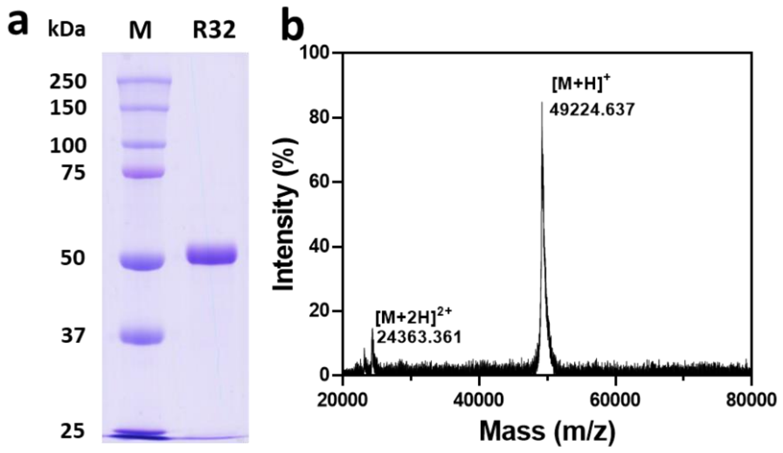

Supplementary Figure 1. Verification of the purified recombinant R32 protein. a,**b**, Coomassie-stained 10% SDS-PAGE gel analysis (**a**) and MALDI-TOF mass spectra (**b**) of the protein. Protein molecular weight marker (M) is included and shown in kDa, and the uncropped gel is shown at the end of this Supplementary Information file. The mass spectrum shows a peak at 49224.64 Da, which is within 0.42% difference of the theoretical value of 49429.91 Da. Data in **a** and **b** are representative of two independent experiments. Source data are provided as a Source Data file.

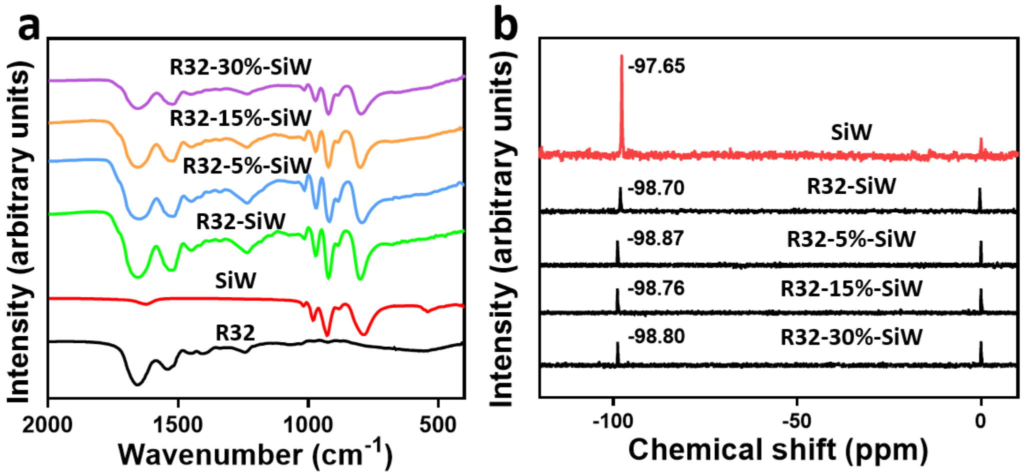

Supplementary Figure 2. Chemical characterizations of SiW complexed R32 hydrogels. R32-SiW, R32-5%-SiW, R32-15%-SiW and R32-30%-SiW correspond to the hydrogels with 0, 5, 15 and 30% di-tyrosine crosslinking degree, respectively. **a**, FTIR spectra of the lyophilized hydrogels in the range of 2000−400 cm^{-1}, with SiW and R32 protein as controls. These data were acquired on a Nicolet 6700 spectrometer (Thermo Fisher Scientific Inc.) equipped with a deuterated triglycine sulfate detector in transmission mode at room temperature. **b**, ^{183}W NMR spectra of SiW alone and the lyophilized hydrogels dissolved in DMSO-d_6. These data were acquired on an Avance III 400 MHz spectrometer (Bruker), with 2 M Na_2WO_4 solution in D_2O as an external reference (the signal at 0 ppm). Data in **a** and **b** are representative of two independent experiments. Source data are provided as a Source Data file.

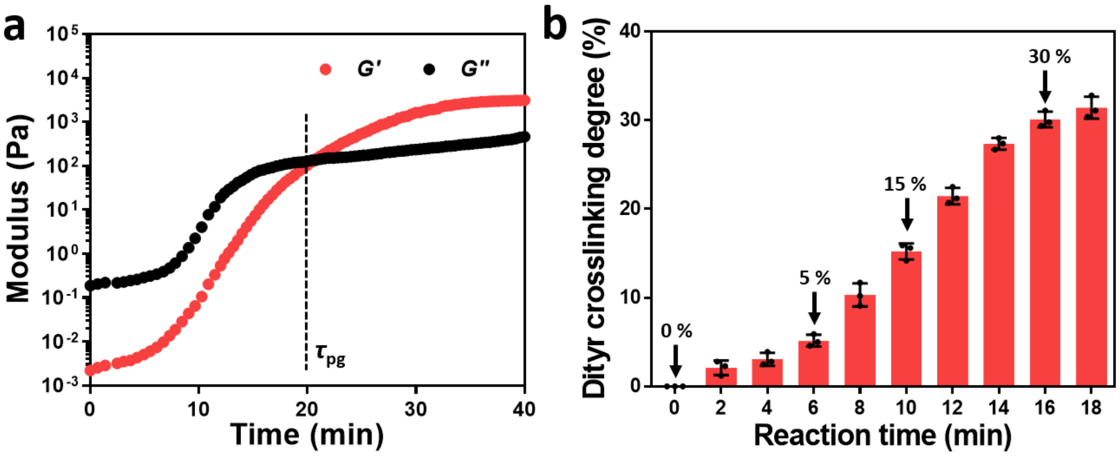

Supplementary Figure 3. Horseradish peroxidase (HRP)-catalyzed di-tyrosine crosslinking of R32 protein. a, Time sweep rheology analysis of a freshly prepared solution containing R32 protein at a final concentration of 20% (w/v), HRP (final enzyme activity of 200 U mL^{-1}), and 0.01% w/w hydrogen peroxide. The dashed line indicates the elapsed time defined as τ_{pg} when G' equals and starts to exceed G'' to enable the formation of a chemical gel, which was determined to be approximately 20 min. **b**, Degree of the di-tyrosine crosslinking for the SiW-complexed R32 hydrogels fabricated with R32 protein solutions undergoing different time periods of pre-crosslinking. The gels with a crosslinking degree of 0, 5%, 15% and 30% were selected for extensive studies. Data in **a** and **b** are representative of two independent experiments. Data in **b** are presented as mean ± s.d. of $n = 3$ independent samples, with individual data points shown as black dots. Source data are provided as a Source Data file.

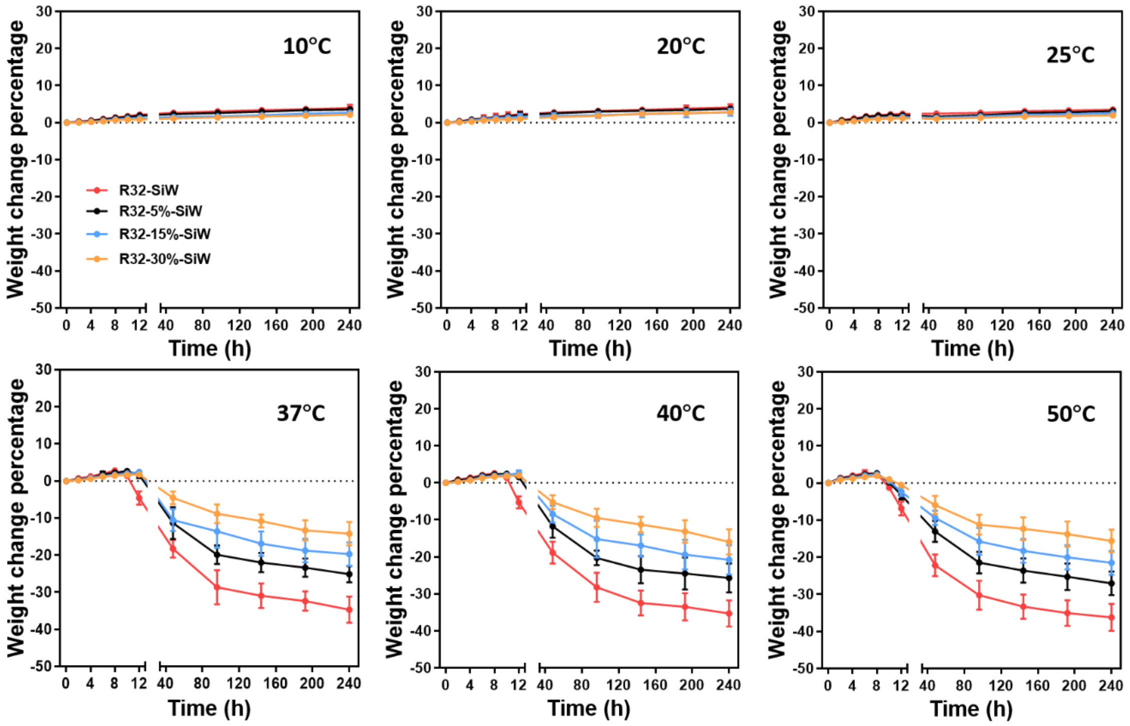

Supplementary Figure 4. Stability and degradation test of the hydrogels in water. Four types of the R32 protein hydrogels with varying degrees of di-tyrosine crosslinking were immersed in deionized water and incubated at the indicated temperatures over an extended time period of 240 h. The time courses of the gel weight change percentages are shown. Data are presented as mean ± s.d. of $n = 3$ independent samples, and are representative of two independent experiments. Source data are provided as a Source Data file.

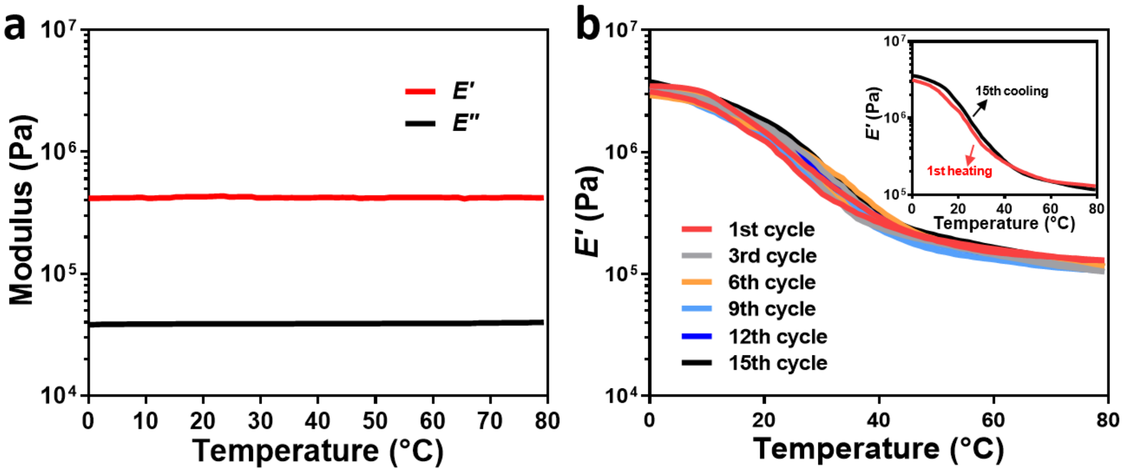

Supplementary Figure 5. Dynamic mechanical analysis (DMA) of the hydrogels. a, The storage (E') and loss (E'') modulus of the chemically crosslinked R32 hydrogel as a function of temperature from 0 to 80 °C at a heating rate of 3 °C min^{-1}. **b**, The storage (E') modulus of the R32-5%-SiW hydrogel as a function of temperature in the cyclic DMA measurements, revealing that the gel maintained the reversibility of E' for at least 15 heating-cooling cycles. Data in **a** and **b** are representative of two independent experiments. Source data are provided as a Source Data file.

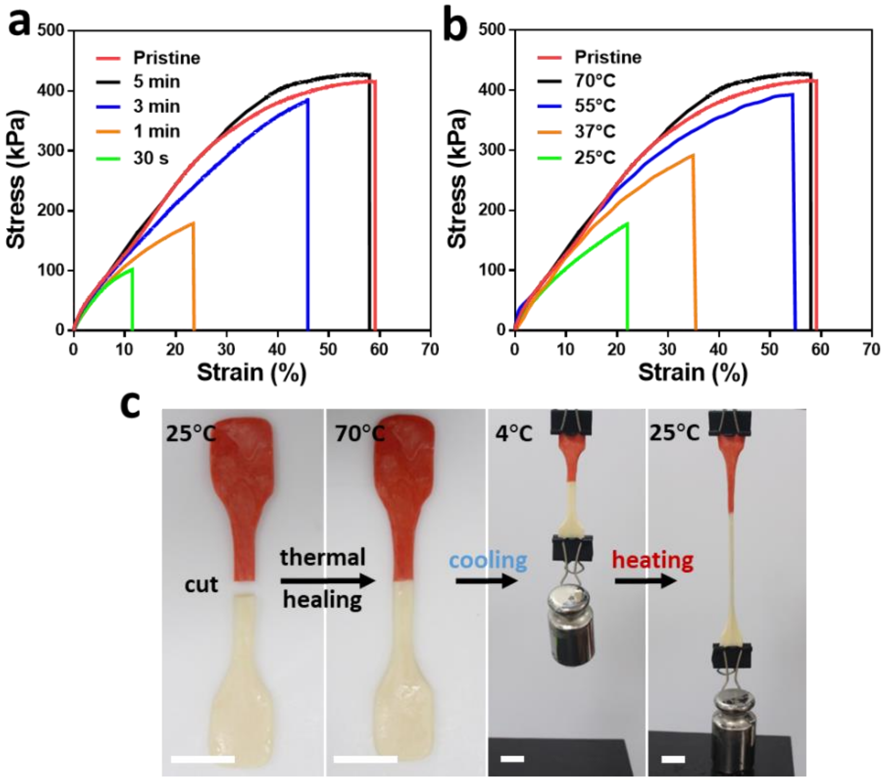

Supplementary Figure 6. Thermal healing of R32-5%-SiW hydrogel. a, Representative tensile stress-strain curves of the R32-5%-SiW hydrogels before and after healing at 70 °C for different time periods. **b**, Representative tensile stress-strain curves of R32-5%-SiW hydrogels before and after healing at different temperatures for 5 min. **c**, Demonstration of temperature-triggered switchable mechanical properties of the thermally healed R32-5%-SiW hydrogel. Scale bar, 8 mm. It should be noted that the pristine dumbbell-shaped hydrogels were cut into two halves, and gently transferred back into the original dumbbell mold for thermal healing without the application of external stress. Tensile testing of the pristine and healed gels were similarly performed at 25 °C. Data in **a**-**c** are representative of two independent experiments. Source data are provided as a Source Data file.

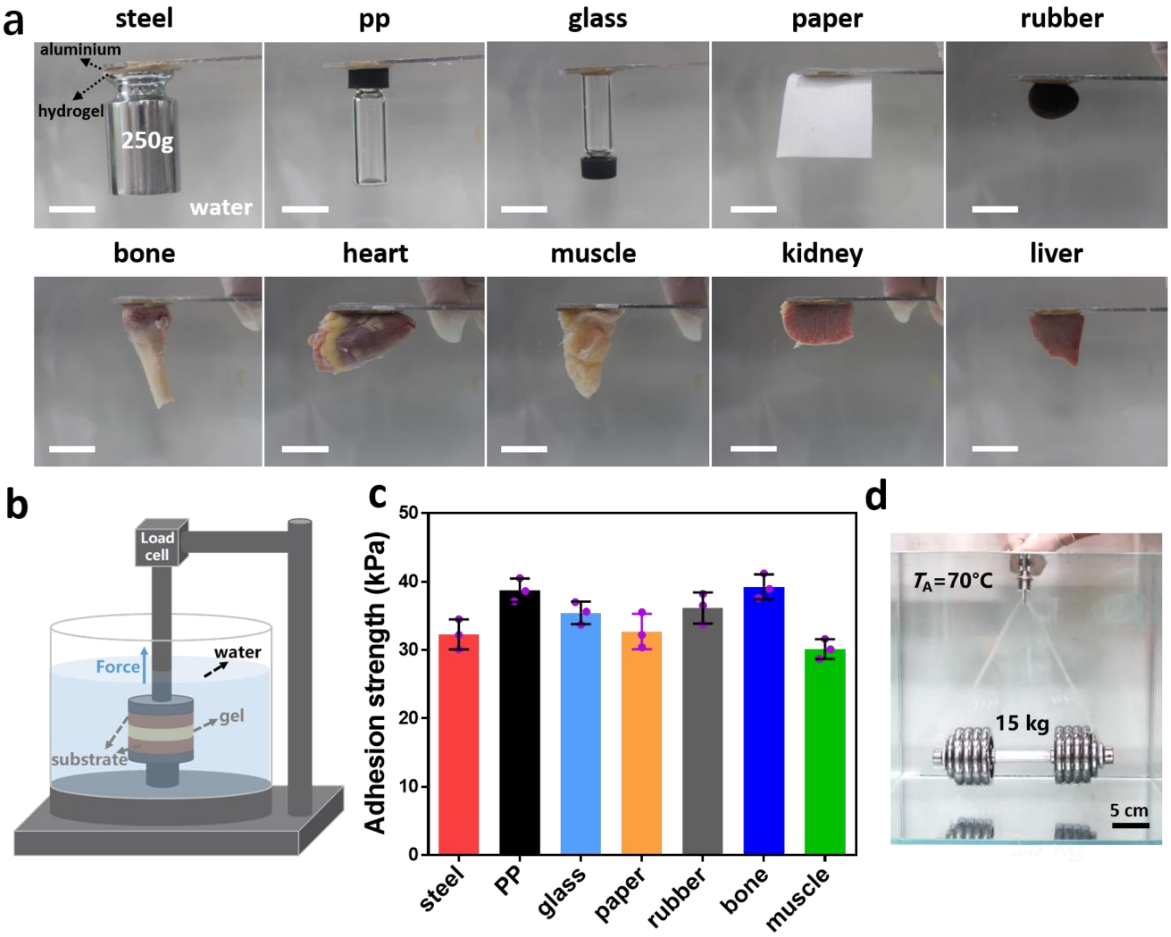

Supplementary Figure 7. Underwater adhesive properties of the R32-5%-SiW hydrogel. **a**, The hydrogel could in-situ adhere to various material surfaces and tissues in water. Scale bar, 10 mm. **b**, Schematic illustration of the probe-tack apparatus used to measure the underwater adhesion of the hydrogels. **c**, The underwater adhesive strength of the R32-5%-SiW hydrogel to diverse surfaces measured by probe-tack tests. For parallel comparison, the same compressive stress (~6 kPa) was applied in all the tests. The data represent the average of three replicates, and error bars correspond to the standard deviations. **d**, The R32-30%-SiW hydrogel is adhesive enough to hang up a weight of 15 kg at 20 °C, after attaching to the steel surface (adhesion area, 3.14 cm^2) in water at an attaching temperature (T_A) of 70 °C. Data in **a**, **c** and **d** are representative of two independent experiments. Data in **c** are presented as mean ± s.d. of $n = 3$ independent samples, with individual data points shown as purple dots. Source data are provided as a Source Data file.

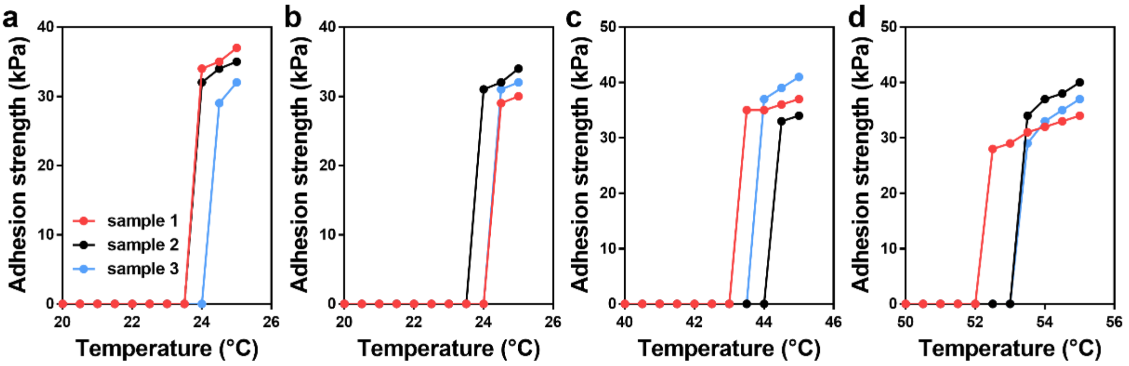

Supplementary Figure 8. Tunability of hydrogel T_i, the temperature at which the hydrogel initiated adhesion. a-d, The R32-SiW (**a**), R32-5%-SiW (**b**), R32-15%-SiW (**c**) and R32-30%-SiW (**d**) hydrogels were subjected to the probe-tack tests using stainless steel as a model substrate, and the underwater adhesion strength was measured for hydrogel triplicates with a temperature interval of 0.5 ℃, representing tenfold higher resolution than the testing in Figure 3a. Data in **a-d** are representative of two independent experiments. Source data are provided as a Source Data file.

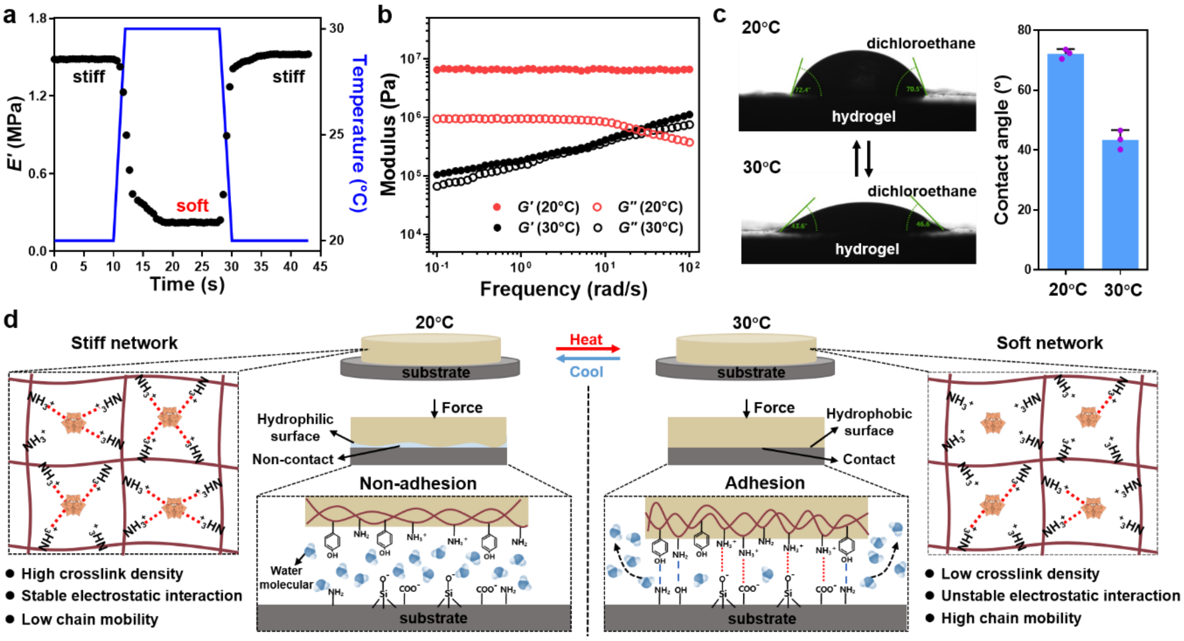

Supplementary Figure 9. Switch of the hydrogel between soft adhesive and stiff non-adhesive states. a, Time sweeps of the storage modulus (*E′*) of the R32-5%-SiW hydrogel upon switching the temperature between 20 °C and 30 °C. **b**, Rheological frequency sweeps of the R32-5%-SiW hydrogel with a constant strain of 0.3% at 20 °C and 30 °C, respectively. **c**, Oil contact angle analysis of the underwater R32-5%-SiW hydrogel at 20 °C and 30 °C, respectively. **d**, Schematic illustration of the proposed switching mechanisms. Data in **a-c** are representative of two independent experiments. Data in **c** (*right*) are presented as mean ± s.d. of $n = 3$ independent samples, with individual data points shown as purple dots. Source data are provided as a Source Data file.

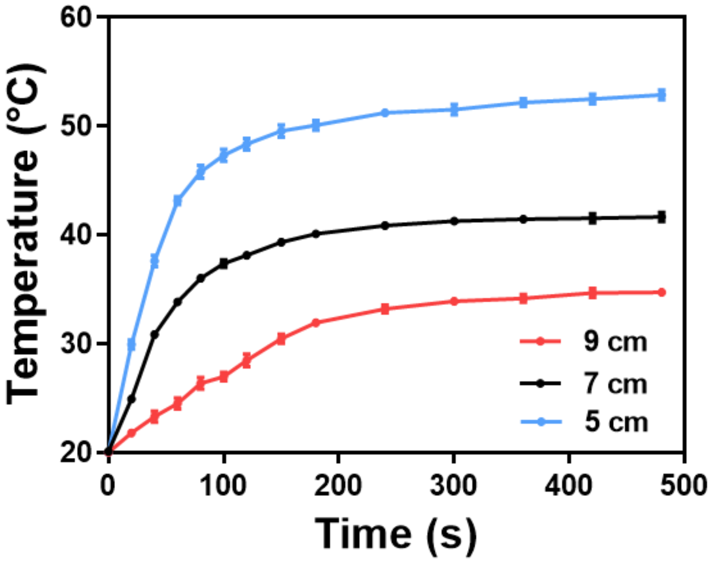

Supplementary Figure 10. Evaluation of the photothermal properties of the M-R32-5%-SiW hydrogel. Evolution of temperature of the underwater disc-shaped gel was monitored upon IR light illumination, from varied distances between the light bulb and gel specimens as indicated in the figure. Data are presented as mean ± s.d. of $n = 3$ independent samples, and are representative of two independent experiments. Source data are provided as a Source Data file.

Uncropped gel 1

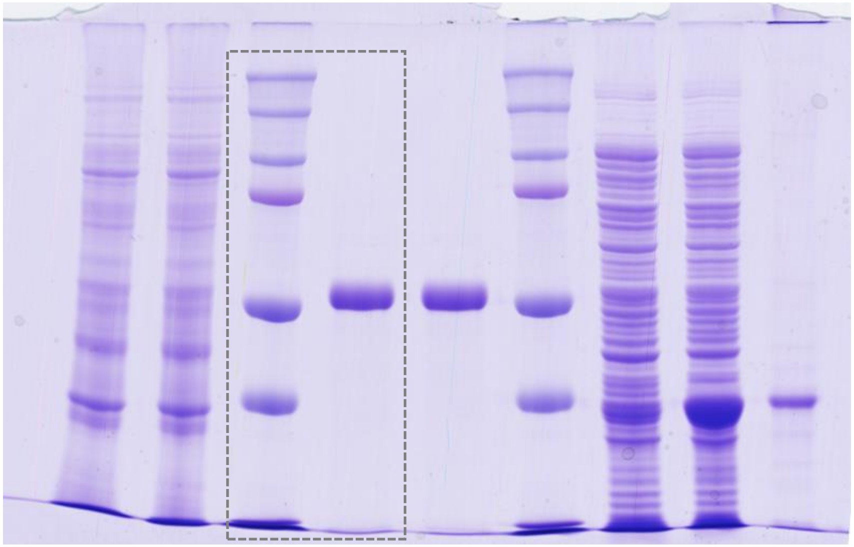

The uncropped scan of the SDS-PAGE gel shown in Supplementary Figure 1a.
