# Supplementary material for: Programmable adhesion and morphing of protein hydrogels for underwater robots
Source: Nat Commun. 2024 Jan 3;15:195. doi: 10.1038/s41467-023-44564-6 (PMC10764313; doi:10.1038/s41467-023-44564-6)
Supplement: Supplementary file 3 — Description of Additional Supplementary Files [file 41467_2023_44564_MOESM3_ESM.pdf]

Description of Additional Supplementary Files

File Name: Supplementary Movie 1
Description: The complexed hydrogel can switch instantly between rigid state (20°C) and soft state (30°C).

File Name: Supplementary Movie 2
Description: Infrared (IR) light-triggered dynamic underwater adhesion of the M-R32-5%-SiW hydrogel.

File Name: Supplementary Movie 3
Description: The chameleon tongue inspired (CTI) robot capturing a prey from a distance in water under cooperative application of an IR light illumination and a magnetic field.
